# CD47 blockade ameliorates autoimmune vasculitis via efferocytosis of neutrophil extracellular traps

**DOI:** 10.1172/jci.insight.167486

**Published:** 2023-08-08

**Authors:** Satoka Shiratori-Aso, Daigo Nakazawa, Takashi Kudo, Masatoshi Kanda, Yusho Ueda, Kanako Watanabe-Kusunoki, Saori Nishio, Sari Iwasaki, Takahiro Tsuji, Sakiko Masuda, Utano Tomaru, Akihiro Ishizu, Tatsuya Atsumi

**Affiliations:** 1 Department of Rheumatology, Endocrinology, and Nephrology, Faculty of Medicine and Graduate School of Medicine, Hokkaido University, Sapporo, Japan.; 2 Division of Rheumatology and Clinical Immunology, Sapporo Medical University, Sapporo, Japan.; 3 Department of Pathology, Sapporo City General Hospital, Sapporo, Japan.; 4 Department of Medical Laboratory Science, Faculty of Health Sciences, Hokkaido University, Sapporo, Japan.; 5 Department of Pathology, Faculty of Medicine and Graduate School of Medicine, Hokkaido University, Sapporo, Japan.

**Keywords:** Autoimmunity, Nephrology, Vasculitis

## Abstract

Neutrophil extracellular trap (NET) formation contributes to immune defense and is a distinct form of cell death. Excessive NET formation is found in patients with anti–neutrophil cytoplasmic antibody–associated (ANCA-associated) vasculitis (AAV), contributing to disease progression. The clearance of dead cells by macrophages, a process known as efferocytosis, is regulated by the CD47-mediated “don’t eat me” signal. Hence, we hypothesized that pathogenic NETs in AAV escape from efferocytosis via the CD47 signaling pathway, resulting in the development of necrotizing vasculitis. Immunostaining for CD47 in human renal tissues revealed high CD47 expression in crescentic glomerular lesions of patients with AAV. In ex vivo studies, ANCA-induced netting neutrophils increased the expression of CD47 with the reduction of efferocytosis. After efferocytosis, macrophages displayed proinflammatory phenotypes. The blockade of CD47 in spontaneous crescentic glomerulonephritis-forming/Kinjoh (SCG/Kj) mice ameliorated renal disease and reduced myeloperoxidase-ANCA (MPO-ANCA) titers with a reduction in NET formation. Thus, CD47 blockade would protect against developing glomerulonephritis in AAV via restored efferocytosis of ANCA-induced NETs.

## Introduction

Necrosis, which is a form of cell death resulting in leakage of cellular contents, induces inflammatory response (1). Dead or dying cells are removed by macrophages through a phagocytic process known as efferocytosis, and CD47 acts as a regulator of this procedure (2). CD47 is a widely expressed cell surface protein and serves as the “don’t eat me” signal or a “marker of self.” The binding of CD47 to signal regulatory protein α (SIRPα) on macrophages inhibits phagocytosis (3). Apoptotic cells are efficiently engulfed by macrophages through the exposure of phosphatidylserine as the “eat me” signal and the downregulation of CD47 as the “don’t eat me” signal (4). Tumor cells and atherosclerotic necrotic cores with increased CD47 expression escape efferocytosis, contributing to disease progression (5, 6). Recently, the involvement of CD47 in autoimmune diseases, including systemic lupus erythematosus (SLE), has been described (7).

Anti–neutrophil cytoplasmic antibody–associated (ANCA-associated) vasculitis (AAV) is a group of disorders associated with systemic small-vessel vasculitis (8, 9). Renal disease in AAV is characterized by necrotizing crescent glomerulonephritis. Crescents are formed by the infiltrating immune and local necrotic cells (10). In particular, ANCA and humoral factors influence neutrophils to induce neutrophil extracellular trap (NET) formation, promoting vascular injury and activation of autoimmunity (11, 12). NETs contribute to immune defense against invading pathogens, with neutrophil death as NETosis (13). However, dysregulated NETs cause excessive tissue damage via release of reactive oxygen species and damage-associated molecular patterns (DAMPs) (1, 12). Moreover, persistent NETs can result in a breakdown of immunotolerance to NET-derived antigens. Therefore, NETs require a clearance system. For example, phorbol myristate acetate–induced (PMA-induced) NETs, which represent a well-characterized NETosis model, are known to be properly digested by deoxyribonuclease I and phagocytosis (14–16).

Here, we hypothesized that ANCA-induced NETs in AAV escape efferocytosis via the upregulation of CD47 and that persistent NETs amplify inflammatory reactions in the disease. We investigated the involvement of CD47 in crescentic glomerulonephritis using human renal tissues from patients with AAV and performed a series of in vitro experiments to examine the effects of CD47 blockade on the efferocytosis of NETs. Finally, we evaluated the therapeutic effects of anti-CD47 antibody in a spontaneous AAV mouse model.

## Results

### Enhanced expression of CD47 in crescentic glomerulonephritis of patients with AAV.

**Figure 1:**
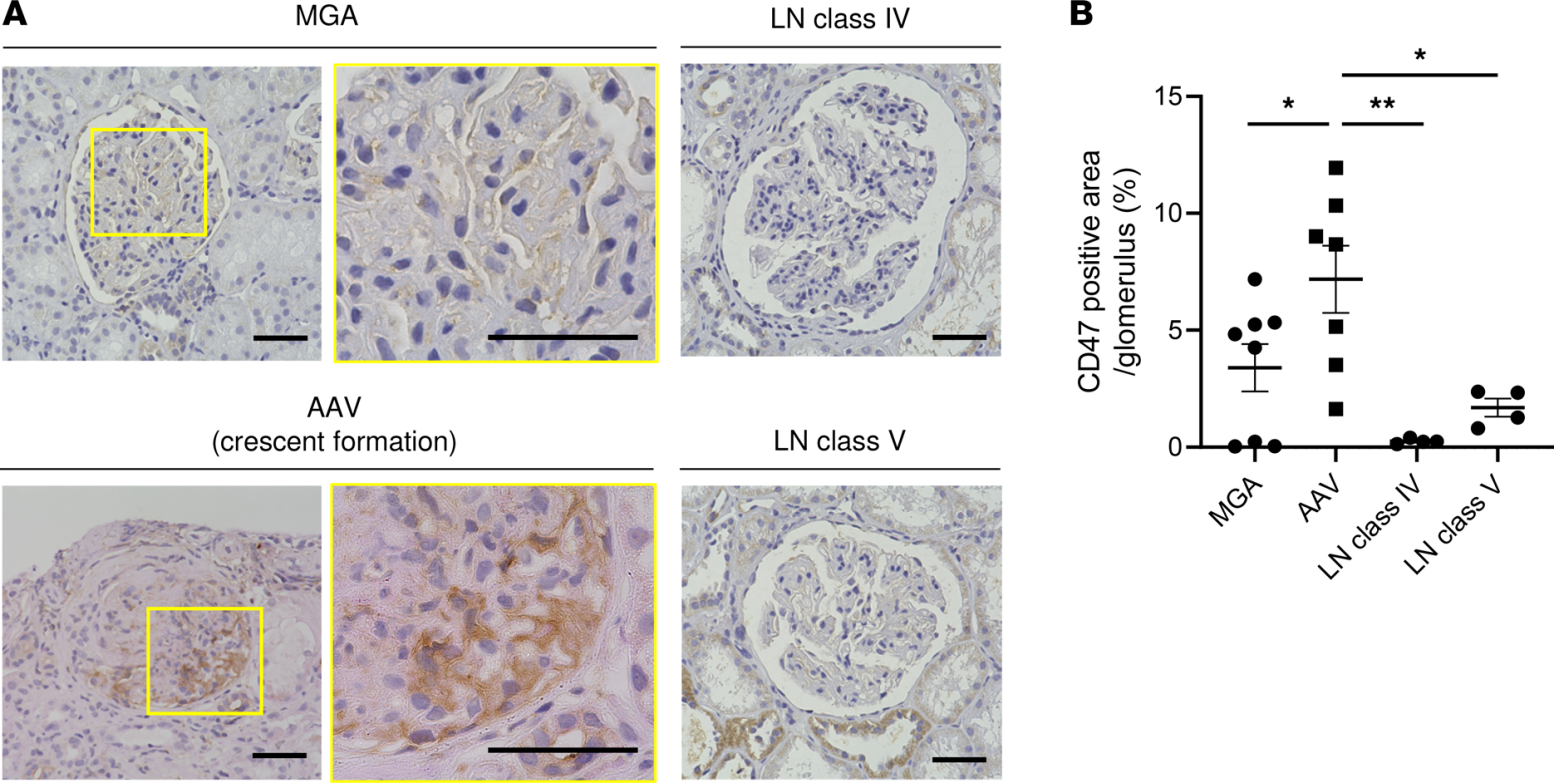
CD47 expression on human renal tissue. (**A**) Representative IHC images for CD47 in MGA, AAV, LN class IV, and LN class V patients. Scale bars: 50 μm. (**B**) Quantification of CD47^+^ area of renal biopsy sections from patients with MGA (*n* = 8), AAV (*n* = 7), LN class IV (*n* = 4), and LN class V (*n* = 4) as a percentage of glomerular area. Data are shown as mean ± SD. *P <0.05, **P < 0.01 (1-way ANOVA with post hoc Dunnett’s multiple-comparison test).

To assess the expression of CD47 in the kidney, we first performed IHC staining for CD47 in human renal biopsy specimens from patients active AAV and other control diseases including lupus nephritis (LN) class IV, LN class V, and minor glomerular abnormalities (MGA) as a control. The clinical characteristics of the patients are presented in Supplemental Table 1 (supplemental material available online with this article; https://doi.org/10.1172/jci.insight.167486DS1). CD47 expression was significantly upregulated in the renal tissues of patients with AAV compared with those of patients with LN and MGA. Immunostaining showed remarkable CD47 positivity in glomerular crescentic lesions, particularly in infiltrated granulocytes and injured epithelium (Figure 1, A and B).

### High expression of CD47 in ANCA-induced netting neutrophils.

**Figure 2:**
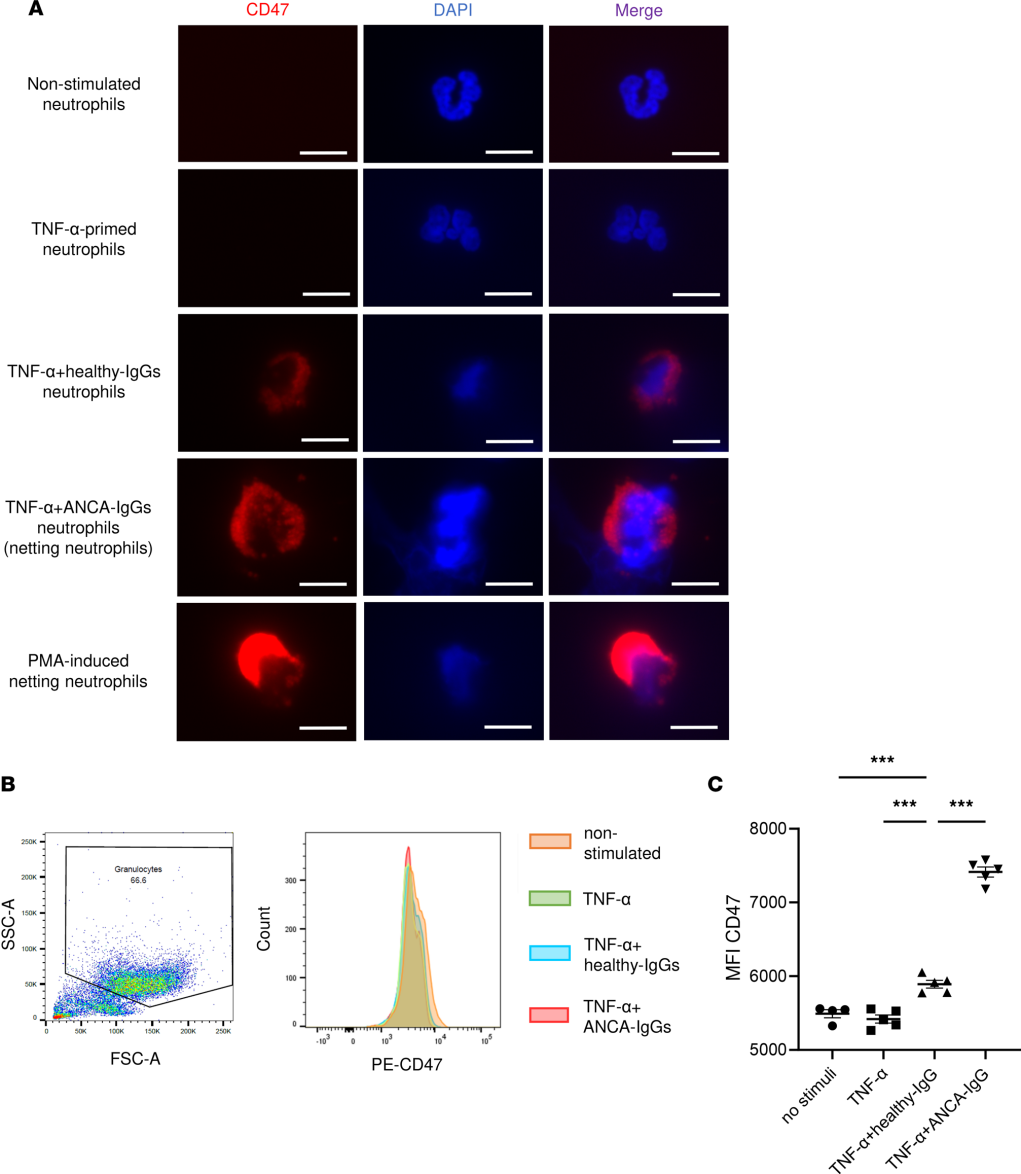
CD47 expression on human neutrophils. Nonstimulated, TNF-α–primed neutrophils incubated with healthy-IgGs or ANCA-IgGs and PMA-induced netting neutrophils were examined. (**A**) Representative images of CD47 and DAPI staining of neutrophils. Red indicates CD47; blue indicates DAPI staining. Scale bars: 10 μm. (**B**) Representative FCM plots of the gating strategy and histogram of CD47 expression on neutrophils. (**C**) Mean fluorescence intensity of CD47 in **B**. Data are shown as mean ± SEM. ***P < 0.001 (1-way ANOVA with post hoc Dunnett’s multiple-comparison test).

Next, we assessed the expression of CD47 on human nonstimulated neutrophils, apoptotic neutrophils, PMA-induced NETs, and ANCA-induced NETs. Ex vivo NET formation was induced by human TNF-α and ANCA-IgGs from patients with myeloperoxidase-ANCA^+^ (MPO-ANCA^+^) AAV. TNF-α–primed neutrophils incubated with or without IgGs from healthy volunteers were used as controls. Neutrophils were treated with staurosporine (STS), an inducer of apoptosis, and the TUNEL assay revealed increased apoptotic neutrophils with condensed nuclei (Supplemental Figure 1, A and B). Immunofluorescence images revealed that ANCA-IgG or healthy-IgG–treated neutrophils primed with TNF-α, and PMA-induced netting neutrophils expressed high levels of CD47, whereas the CD47 expression levels on nonstimulated, apoptotic, and TNF-α–primed neutrophils were low (Figure 2A and Supplemental Figure 1, C and D). Increased CD47 expression was observed on the cell membrane of neutrophils with ANCA- and PMA-induced NETs but not on extracellular DNA fibers (Supplemental Figure 1E and Supplemental Video 1). Flow cytometry (FCM) analysis showed that healthy-IgG–treated neutrophils primed with TNF-α expressed significantly higher levels of CD47 than nonstimulated neutrophils and that the expression of CD47 on ANCA-induced netting neutrophils was significantly higher than that on neutrophils treated with healthy-IgGs (Figure 2, B and C).

### Macrophage efferocytosis of ANCA-induced NETs via CD47 signaling.

**Figure 3:**
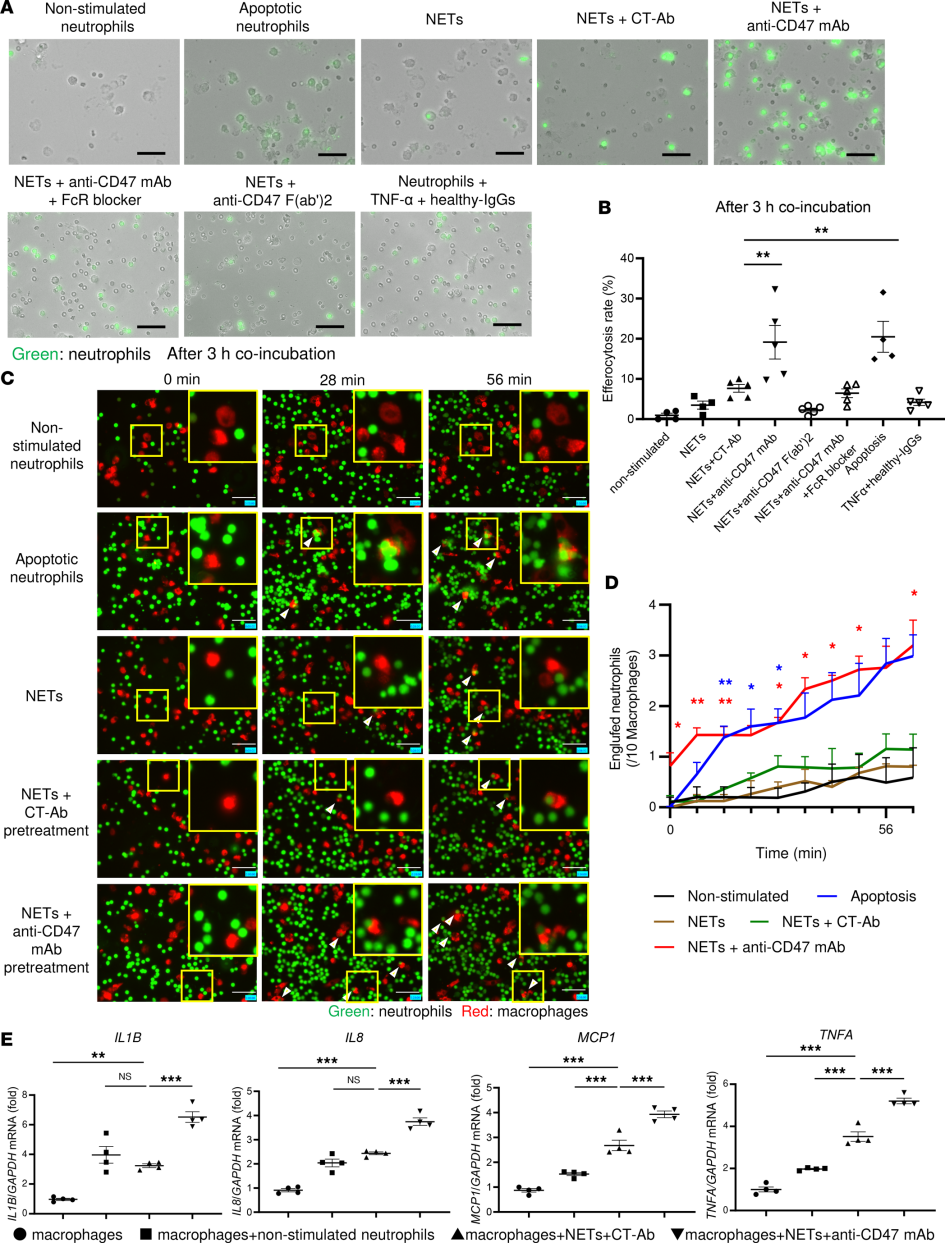
Efferocytosis of human neutrophils via CD47 signaling. (**A**) Representative images of macrophages after 3 hours of incubation with CMFDA-labeled nonstimulated, apoptotic neutrophils, neutrophils incubated with TNF-α + healthy-IgGs, ANCA-induced NETs, and NETs treated with CT-Ab, anti-CD47 mAb, anti-CD47 mAb + FcR blocker, or anti-CD47 F(ab’)2 fragments. Cocultures were rinsed before imaging. Green indicates neutrophils. Scale bars: 50 μm. (**B**) Efferocytosis rate (the percentage of CMFDA^+^ macrophages) in **A**. (**C**) Representative time-lapse images of efferocytosis assay of neutrophils. Green indicates neutrophils, red indicates macrophages. Scale bars: 50 μm. Arrowheads indicate the engulfed neutrophils by macrophages. (**D**) Quantification of the number of engulfed nonstimulated (*n* =3), apoptotic neutrophils (*n* =3), NETs (*n* =2), and NETs treated with CT-Ab (*n* =3) or anti-CD47 mAb (*n* =3) every 7 minutes. Statistical analysis was performed using 1-way ANOVA, followed by Dunnett’s multiple-comparison test compared with NETs treated with CT-Ab. (**E**) mRNA expressions of *IL1B*, *IL8*, *MCP1*, and *TNFA* by efferocytosis. Total RNA was extracted from macrophages after 2 hours of their exposure to nonstimulated neutrophils, NETs treated with CT-Ab or anti-CD47 mAb, and monocultured macrophages (*n* =4 for each). Data are shown as mean ± SEM. *P <0.05, ** *P* <0.01, ***P <0.001 (1-way ANOVA with post hoc Dunnett’s multiple-comparison test).

Based on human and ex vivo data, we hypothesized that enhancing the expression of CD47 on injured cells, including ANCA-induced NETs, initiates escape from efferocytosis. The 5-chloromethylfluorescein diacetate–labeled (CMFDA-labeled) human neutrophils were induced into STS-treated apoptotic neutrophils, TNF-α + healthy-IgG–treated neutrophils, or TNF-α + ANCA–treated NETs. Apoptotic neutrophils and NET neutrophils were cocultured with M1 macrophages in the presence of pretreatment with PBS, mouse IgG1 isotype control Ab (CT-Ab), anti-CD47 mAb, anti-CD47 mAb + Fc receptor (FcR) blocker, or anti-CD47 F(ab’)2 fragments. Phase and fluorescence imaging showed that, after 3 hours of coculture, nonstimulated neutrophils and TNF-α + healthy-IgG–treated neutrophils escaped efferocytosis. Apoptotic neutrophils were engulfed more efficiently by macrophages than NETs (efferocytosis rate/apoptotic neutrophils + macrophages, 20.5% ± 3.8%; NETs pretreated with CT-Ab + macrophages, 7.7% ± 2.2%; *P* < 0.05), whereas the efferocytosis rate of NETs was significantly increased by pretreatment with anti-CD47 mAb (NETs pretreated with anti-CD47 mAb + macrophages [19.1% ± 4.2%, *P* < 0.05] compared with NETs pretreated with CT-Ab + macrophages) (Figure 3, A and B). The efferocytosis rate of NETs was not recovered by pretreatment with a combination of anti-CD47 mAb and FcR blocker or anti-CD47 F(ab’)2 fragments. To precisely evaluate cellular dynamics during efferocytosis, time-lapse imaging was performed using CMFDA-labeled neutrophils (green) and PKH26 labeled macrophages (red) (Figure 3C). Fluorescence images of cocultures were captured every 7 minutes for 1 hour from the beginning of the coculture. NETs were not engulfed by macrophages, but treatment with anti-CD47 mAb increased the engulfed NETs by macrophages, similar to the apoptotic neutrophils (Figure 3, C and D, and Supplemental Videos 2–6). In immunostaining to determine the clearance of NETs by efferocytosis, unengulfed NETs (citrullinated histone H3 [citH3] +/PKH26^+^) were reduced by anti-CD47 mAb treatment (residual rate/NETs + macrophages, 86.7% ± 18.9%; NETs pretreated with CT-Ab + macrophages, 84.3% ± 11.2%; NETs pretreated with anti-CD47 mAb, 42.4% ± 10.7%; *P* < 0.05) (Supplemental Figure 2, A and B).

To evaluate the effect of CD47 on ANCA-induced NET formation, NETs were pretreated with anti-CD47 mAb or CT-Ab. SYTOX Green staining showed that ANCA-induced NET formation was not affected by CD47 blockade (Supplemental Figure 2, C and D), indicating that CD47 blockade initiated the clearance of NETs via efferocytosis but did not directly affect NET formation. To assess the response of macrophages during efferocytosis of ANCA-induced NETs, total RNA was extracted from macrophages after 2 hours of exposure to neutrophils. In macrophages, coincubation with NETs enhanced the mRNA expression of proinflammatory cytokines (*IL1B*, *IL8*, monocyte chemotactic protein-1 [*MCP1*], and *TNFA*), and treatment with anti-CD47 mAb further increased these expression levels (Figure 3E).

### Efferocytosis of injured endothelium via CD47 signaling.

Injured vascular endothelium is a part of crescentic glomerular lesions in AAV. To investigate the role of efferocytosis of necrotic endothelial cells, we performed an efferocytosis assay using CMFDA-labeled human umbilical vein endothelial cells (HUEhT) (green) and PKH26-labeled macrophages (red) (Supplemental Figure 3C). Reactive oxygen species released from NETs result in injury of vascular endothelial cells. Thus, HUEhT cells were treated with 500 μM hydrogen peroxide (H_2_O_2_) to induce necrosis. The necrosis was confirmed by propidium iodide (PI) staining (Supplemental Figure 3, A and B). Time-lapse imaging showed that necrotic HUEhT escaped macrophage efferocytosis, but necrotic HUEhT pretreated with anti-CD47 mAb were engulfed by macrophages (Supplemental Figure 3, C and D, and Supplemental Videos 7–10).

### Blockade of CD47 improves renal involvement in spontaneous crescentic glomerulonephritis-forming/Kinjoh (SCG/Kj) mice via the restored efferocytosis of NETs.

**Figure 4:**
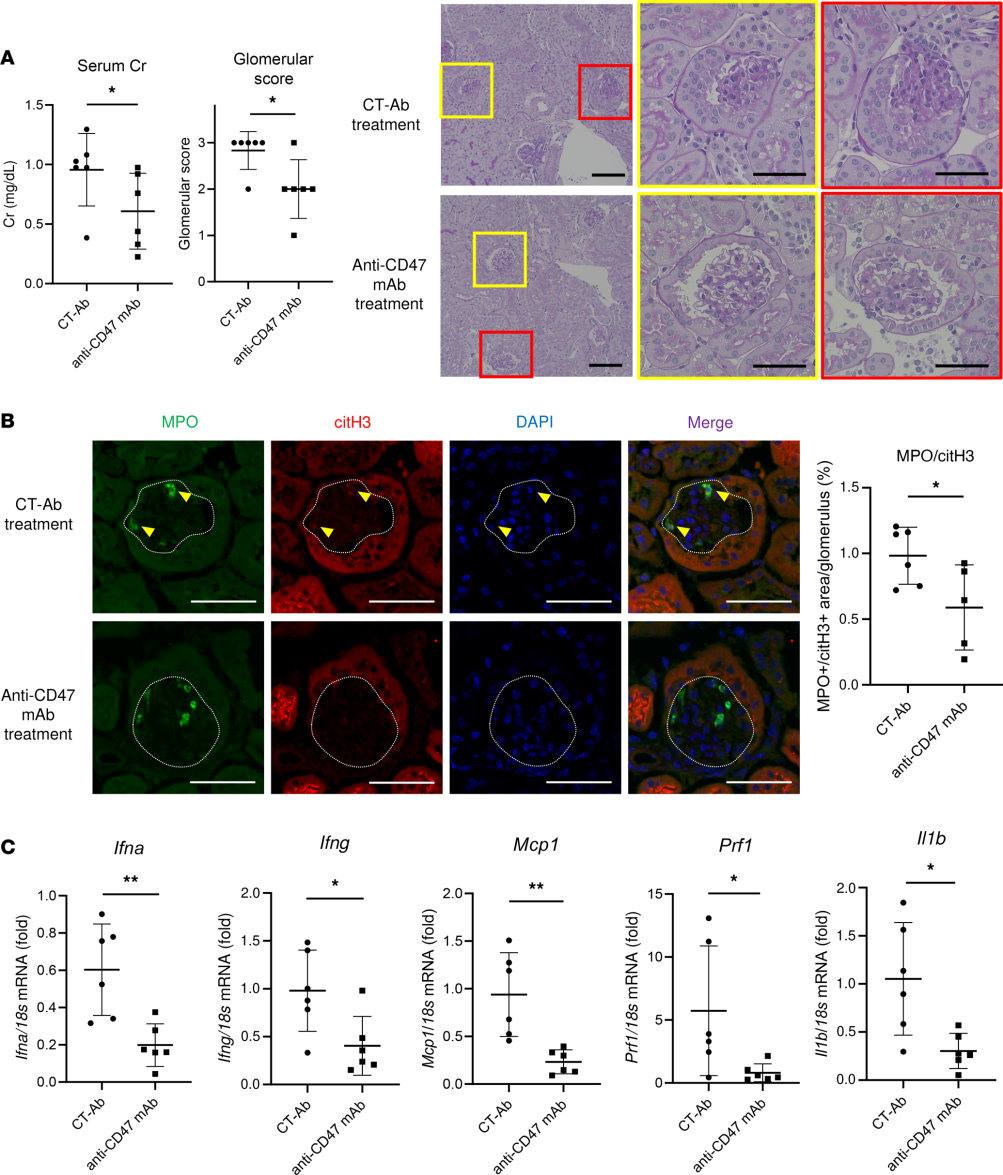
Blockade of CD47 protected mice from spontaneous development of vasculitis. Eight-week-old SCG/Kj mice were i.p. injected with CT-Ab or anti-CD47 mAb every 5 days for 2 weeks (*n* =6 for each). (**A**) Results of serological tests (as assessed by Mann-Whitney *U* test) and histopathology. The glomerular activity score was assessed. Scale bars: 100 μm (low magnification) and 50 μm (high magnification). (**B**) Representative images of citH3 staining and quantitative analysis of glomeruli for MPO^+^/citH3^+^ double-positive area. White dotted lines and yellow arrowheads indicated glomeruli and MPO^+^/citH3^+^ double-positive cells, respectively. Green indicates MPO; red indicates citH3; blue indicates DAPI staining. Scale bars: 50 μm. (**C**) mRNA expression of *Ifna*, *Ifng*, *Mcp1*, *Prf1*, and *Il1b* in the whole kidney. Data are shown as mean ± SD. *P <0.05, **P <0.01 (Student’s unpaired *t* test).

Having shown that CD47 blockade ex vivo contributes to the clearance of NETs and injured endothelium via efferocytosis, we tested whether renal disease in AAV is abrogated by the systemic administration of anti-CD47 antibody. SCG/Kj mice develop systemic necrotizing glomerulonephritis with ANCA production (17). Eight-week-old mice were treated with i.p. injections of either anti-CD47 mAb or rat IgG2a isotype CT-Ab every 5 days for 2 weeks, and 10-week-old mice were analyzed. CT-Ab–treated SCG/Kj mice developed glomerulonephritis with renal dysfunction. Treatment with anti-CD47 mAb improved renal failure (serum creatinine: anti-CD47 mAb, 0.96 ± 0.30 mg/dL, versus CT-Ab, 0.61 ± 0.32 mg/dL; *P* <0.05) and attenuated kidney injury in histopathological examination (glomerular score: anti-CD47 mAb, 2.0 ± 0.63, versus CT-Ab, 2.8 ± 0.41; *P* <0.05) (Figure 4A). Ly6B/citH3–double positive NETs were detected in glomeruli of untreated SCG/Kj mice (Supplemental Figure 4A), and the area of MPO and citH3–double positive NETs was decreased in glomeruli of anti-CD47 mAb–treated mice, compared with that of CT-Ab–treated mice (Figure 4B).

Moreover, anti-CD47 mAb reduced the mRNA expression of proinflammatory genes, including *Ifna*, *Ifng*, *Mcp1*, *Prf1*, and *Il1b* in renal tissue (Figure 4C).

We performed RNA-Seq to assess the transcriptome changes in the renal tissue of SCG/Kj mice. Canonical pathway analysis using Ingenuity Pathway Analysis (IPA) software revealed the involvement of immunological responses including ‘Communication between innate and adaptive immune cells’ in CD47 blockade-treated mice (Supplemental Figure 4B). Next, to elucidate the mechanism of the protective effect of CD47 blockade, we tested whether anti-CD47 antibody binds to injured glomerular cells by immunostaining with anti–rat IgG, which is a host species of anti-CD47 mAb. Binding of anti-CD47 mAb was observed in the glomerulus of anti-CD47 mAb–treated SCG/Kj mice, whereas that of anti-CD47 mAb–treated C57BL/6 mice was not detected (Supplemental Figure 4C).

### The immune cell profiles in kidney of SCG/Kj mice treated with CD47 blockade.

**Figure 5:**
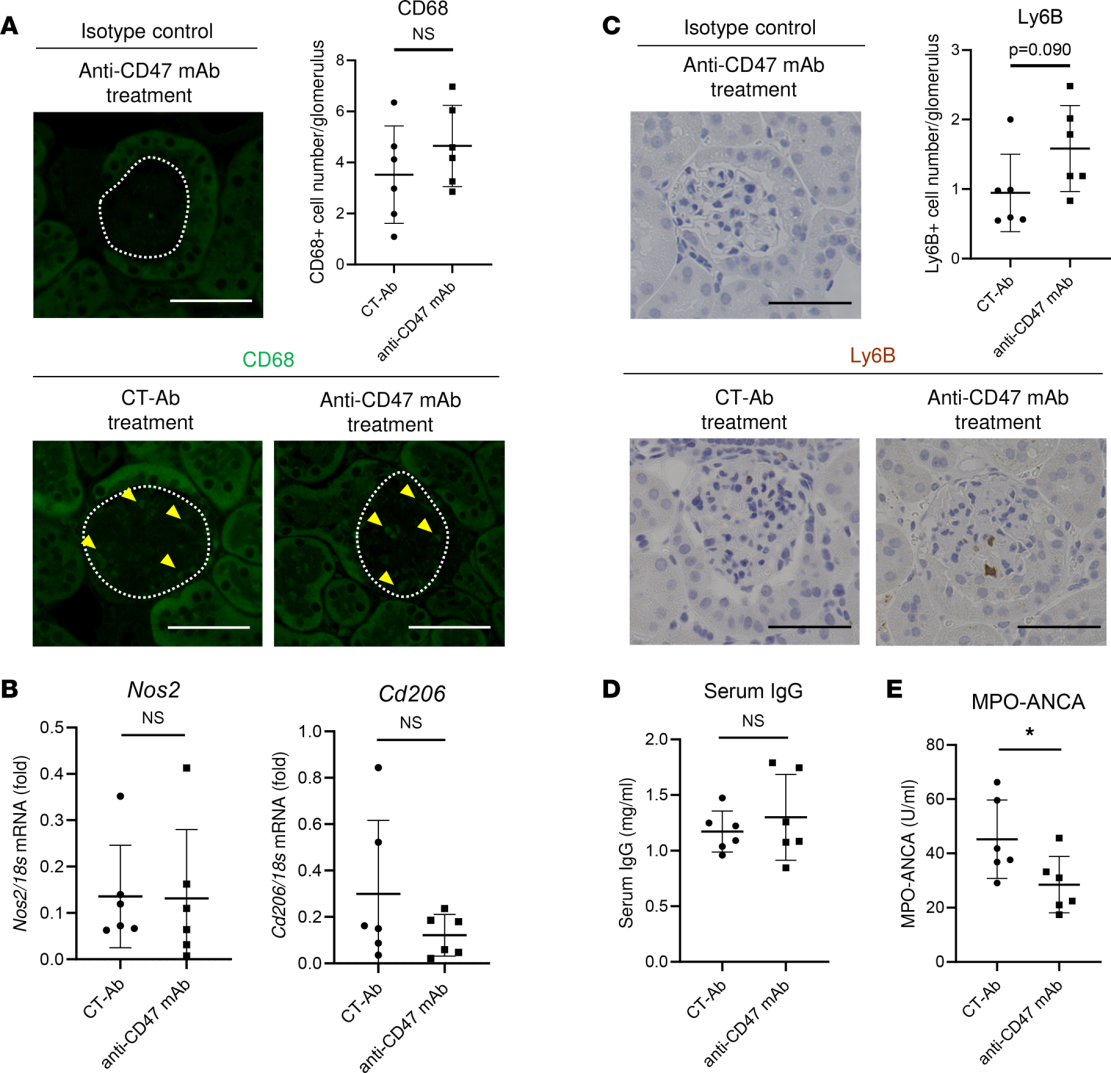
The immune cell profiles of the kidneys and systemic immune responses in SCG/Kj mice treated with CD47 blockade. (**A**) Representative images and quantitative analysis of glomeruli by immunostaining for macrophages. White dotted lines and yellow arrowheads indicate glomeruli and CD68^+^ cells, respectively. Scale bars: 50 μm. (**B**) mRNA expressions of *Nos2* and *Cd206* in whole kidney. (**C**) Representative images and quantitative analysis of glomeruli by immunostaining for neutrophils. Scale bars: 50 μm. (**D**) The results of serum IgG. (**E**) The results of serum MPO-ANCA titer. Data are shown as mean ± SD. *P <0.05 (Student’s unpaired *t* test).

Considering ex vivo findings that macrophages engulf necrotic cells through the effects of CD47 blockade, but macrophages show proinflammatory properties, we questioned the mechanism how CD47 blockade influences the dynamics of immune cells in the kidneys of SCG/Kj mice. Immunostaining showed that the number of glomerular infiltrating CD68^+^ macrophages in SCG/Kj mouse kidneys was not influenced by CD47 blockade (Figure 5A). Moreover, *Nos2* (M1 macrophage marker) and *Cd206* (M2 macrophage marker) mRNA expression in kidneys treated with CD47 blockade was similar to the expression in those treated with CT-Ab (Figure 5B). In mice treated with CD47 blockade, the glomerular infiltrating Ly6B^+^ neutrophils showed an increasing trend (Figure 5C), implying that activated macrophages exhibiting efferocytosis may promote the recruitment of immune cells beyond those needed for focal cellular waste disposal. Furthermore, we performed deconvolution of our bulk kidney RNA-Seq data using a reference single-cell RNA-Seq data (18) to estimate the immune cell ratio in our data. The deconvolution revealed that CD47 blockade did not alter the profiles of immune cells, including neutrophils, macrophages, and lymphocytes in the kidney (Supplemental Figure 4D), and this was consistent with the histological findings.

### Systemic immune responses in SCG/Kj mice by CD47 blockade.

Since CD47 blockade promoted immune cell infiltration in the kidneys of SCG/Kj mice, we next evaluated the effect of CD47 blockade on systemic immunity. Treatment with CD47 blockade did not affect total IgG (Figure 5D) but significantly reduced MPO-ANCA (Figure 5E) in the serum of SCG/Kj mice. FCM analysis revealed that there were no significant differences in the number or proportion of splenic cells (neutrophils, monocytes, macrophages, DCs, T cells, and B cells) between anti-CD47 mAb–treated and CT-Ab–treated mice (Supplemental Figure 5, A and B).

## Discussion

Necrotizing vasculitis in AAV is formed by the necrosis of immune cells and blood vessels (10). We showed efferocytosis as a therapeutic target for AAV to treat necrotic lesions. Therefore, we demonstrated several findings. Crescentic glomerular lesions in the kidneys of patients with AAV and ex vivo ANCA-induced NETs increased the expression of CD47, an antiefferocytotic signal. These results indicate that increased expression of CD47 in crescentic glomerulonephritis and ANCA-induced NETs is involved in AAV pathogenesis. In the efferocytosis assay, ANCA-NETs and injured endothelium escaped efferocytosis by macrophages, and the blockade of CD47 restored the efferocytosis of these cells. In vivo, blockade of CD47 by anti-CD47 mAb ameliorated renal injury and inflammation in a spontaneous AAV mouse model via the restored efferocytosis of NETs.

Neutrophils without stimulation store the majority of CD47 in intracellular-specific granules, and chemoattractant-stimulated neutrophils express CD47 to the cell membrane with granular pattern (19, 20). Apoptotic cells are removed by macrophage-mediated efferocytosis through the downregulation of CD47 as a “don’t eat me” signal (2, 4). Tumor and atherosclerotic necrotic cores increase the expression of CD47 and prevent efferocytosis, thereby causing disease progression. Blockade of CD47 prevents tumor growth (3) and atherosclerosis (6). Moreover, treatment with a CD47 blocking antibody ameliorates renal ischemia-reperfusion injury in mice (21). Thus, targeting CD47 is now in the spotlight as a therapy for various diseases involving impaired efferocytosis. Here, blockade of CD47 restored the efferocytosis of NETs and necrotic endothelium in an ex vivo model and protected against renal injury in AAV mice. NETs and necrotic debris themselves serve as DAMPs, initiating surrounding cell injury and amplifying the loop between cell death and inflammation (22); thus, the removal of necrotic contents through efferocytosis might prevent renal disease in AAV. In addition, CD47 blockade reduced pathogenic MPO-ANCA levels in spontaneous AAV mice with a decrease of NETs in glomeruli. The number of splenocytes and total IgG levels were not affected by the CD47 blockade; thus, blockade of CD47 in AAV does not influence systemic immunity but specifically might contribute to the reduction of MPO-ANCA via the restored efferocytosis of NETs containing MPO as an autoantigen. Meanwhile, the migration of neutrophils into the kidneys of AAV mice was enhanced by the CD47 blockade. In vitro, macrophages exhibited a proinflammatory phenotype during efferocytosis of ANCA-NETs via CD47 blockade. In cancer cells, blockade of CD47 actively modulates the immune response and efferocytosis to initiate antitumor cytotoxicity (23, 24). In lymphocytic choriomeningitis virus–infected mice, antibody-mediated blockade of CD47 enhances antigen-presenting cell function and CD8^+^ T cell responses (25). Thus, it is important to understand the mechanism of immune response under the blockade of CD47, particularly in autoimmune diseases. CD47 KO in *Fas^lpr^* lupus mice reduces autoantibodies and protects mice against lupus (26). In experimental autoimmune encephalomyelitis (EAE) mouse models, pharmacological and genetic inhibition of CD47 in the induction phase reduces disease severity (27). In contrast, blocking with anti-CD47 mAb in lupus or EAE with active phase worsens the disease activity (26, 27). The therapeutic effect may be influenced by the timing of CD47 inhibition in the development of autoimmune diseases. In our mouse study, anti-CD47 mAb was administered in the phase of disease development (17); as a result, it prevented renal disease with a reduction in proinflammatory gene expression, whereas it increased infiltrating neutrophils with macrophage activation. Our study revealed the differences in gene expression of proinflammatory cytokines under treatment with CD47 blockade between macrophages coincubated with NETs in vitro (Figure 3E) and the whole kidneys of SCG/Kj mice (Figure 4C). This discrepancy is likely due to several causes; in in vitro conditions, enforced efferocytosis against NETs initiates inflammatory signals in macrophages, but in the injured kidney, necrotic cells are processed by restored efferocytosis, which might ameliorate surrounding tissues damage and reduce the inflammatory gene expressions. These findings indicate that the renoprotective effects of efferocytosis of NETs and glomerular necrotic cells might exceed the activated immune response via CD47 blockade (Supplemental Figure 6). In addition, blockade of CD47 in combination with immunosuppression, including rituximab, is known to be more effective in patients with non-Hodgkin’s lymphoma (28). Previous reports have shown that CD47 blockade induces efferocytosis of lymphoma cells via Fc-FcR interactions (29, 30), and efferocytosis assay of ANCA-induced NETs using FcR blocker or anti-CD47 F(ab’)2 in this study showed that the effects of anti-CD47 mAb on efferocytosis were FcR dependent. Thus, regulating immune response during efferocytosis might boost the effectiveness of CD47 blocking therapy. Further studies are needed in order to understand the immune mechanisms of AAV under CD47 inhibition. There are other limitations to our study. In histological analysis of human renal biopsy, patients with biopsy-proven MGA clinically presented with hematuria or proteinuria; therefore, they might be in subclinical phage of kidney disease, which could influence CD47 expression. Second, the effects of the anti-CD47 mAb on the active phase of AAV were not evaluated. Considering that contradictory effects of CD47 blockade, presumably due to the macrophage activation, have been reported in lupus and EAE mouse models, further studies are needed to assess the therapeutic effects of anti-CD47 mAb in AAV mouse models with active disease.

In conclusion, ANCA-induced NETs in AAV escape efferocytosis via the upregulation of CD47. Blockade of CD47 restores efferocytosis of NETs and injured vascular endothelium, resulting in the improvement of renal disease in AAV. Therefore, CD47 blocking may be a novel therapeutic strategy for AAV.

## Methods

### IHC for CD47 in human renal tissues.

Formalin-fixed, paraffin-embedded renal biopsy tissues from patients with AAV, LN class IV, LN class V, and controls (MGA) were sliced, deparaffinized, and rehydrated with lemosol and ethanol. Antigen retrieval was performed in sodium citrate buffer (10 mM sodium citrate, pH 6) in a microwave. Endogenous peroxidase was blocked with 3% H_2_O_2_ in methanol, and samples were covered with 10% goat serum at room temperature for 1 hour. Slides were incubated at 4°C overnight with anti-CD47 antibody (1:700; HPA044659, Sigma-Aldrich). Biotin-labeled anti–rabbit polyclonal antibodies (426012, Nichirei) were added for 1 hour at room temperature, followed by peroxidase-conjugated streptavidin (Nichirei) and Histofine DAB Substrate Kit (Nichirei). The positive area was quantified using the ImageJ/Fiji software (NIH).

### Induction of apoptosis and NETs in human neutrophils.

Neutrophils were isolated from healthy human donors by density centrifugation at 500*g* for 30 minutes at 20°C using Polymorphprep (Axis-Shield), and were suspended in RPMI 1640 (Sigma-Aldrich). Apoptosis was induced using 0.05 μM STS (FUJIFILM Wako) for 4 hours. Cell apoptosis was assessed by fluorescence microscopy using TUNEL staining kit (Medical & Biological Laboratories [MBL]). For ANCA-NET induction, neutrophils were primed with human TNF-α (5 ng/mL; Merck) for 15 minutes and then exposed to 400 μg IgGs eluted from the serum of healthy volunteers or patients with MPO-ANCA^+^ AAV for 4 hours (2 hours for the efferocytosis assay). For PMA-NET induction, neutrophils were exposed to PMA (50 nmol/L; Merck) for 2 hours. NET formation was quantified based on the SYTOX Green^+^ area of immunostaining as the mean luminance value using ImageJ software. To assess the effects of CD47 blockade on NET induction, neutrophils were exposed to PBS, mouse IgG1 isotype CT-Ab (10 μg/mL; MOPC-21, BioXcell), or anti-CD47 mAb (10 μg/mL; B6H12, BioXcell) for 30 minutes before NET induction.

### Immunofluorescence staining for CD47 of human neutrophils.

Extracted neutrophils were seeded into 8-well chamber slides and stimulated to induce NET formation or apoptosis. Neutrophils were fixed with 4% paraformaldehyde (PFA), covered with 1% BSA at room temperature for 1 hour, and exposed to phycoerythrin (PE) anti-CD47 antibody (1:100; 323108, BioLegend) for 30 minutes. Slides were mounted using VECTASHIELD with DAPI (Vector Laboratories).

### Flow cytometric detection of CD47^+^ neutrophils.

To measure the cell surface expression of CD47, neutrophils were stained with PE anti-CD47 antibody. After filtering out the debris with a mesh, the percolated cells were analyzed using a BD FACS Verse.

### Induction of necrosis in HUEhT cells.

HUEhT cells, obtained from the Japanese Collection of Research Bioresources (JCRB) cell bank (Osaka, Japan), were suspended in HuMedia-EG2 (Kurabo) and seeded into plates at a density of 1 × 10^5^/mL. After overnight culture, HuMedia-EG2 medium was replaced with serum-free DMEM (Sigma-Aldrich). Then, 500 μM H_2_O_2_ was added to induce necrosis for 4 hours. Cell necrosis was assessed by immunofluorescence staining with PI (BD Biosciences). The PI^+^ area of immunostaining was quantified as the mean luminance value using ImageJ software.

### Preparation of F(ab′)2 fragments.

CD47 F(ab’)2 fragments were produced from ficin digest of IgG using Pierce Mouse IgG1 Fab and F(ab’)2 Micro Preparation Kit (Thermo Fisher Scientific), according to the manufacture’s recommendations. The purified anti-CD47 F(ab’)2 fragments were collected for the efferocytosis assay.

### Isolation and phenotype induction of human monocyte–derived macrophages.

PBMCs were isolated from healthy human donors by density centrifugation at 300*g* for 20 minutes at 20°C using Ficoll-Paque Plus (GE Healthcare). Monocytes were isolated from PBMCs using CD14 MicroBeads (Miltenyi Biotech) and magnetic columns, according to the manufacturer’s instructions. After magnetic separation, purified CD14^+^ monocytes were suspended in ImmunoCult-SF Macrophage Medium (Stemcell Technologies) with human recombinant macrophage colony stimulating factor (M-CSF; 5 μg/mL; Peprotech) and seeded into plates at a density of 1 × 10^6^/mL. LPS (10 ng/mL; Sigma-Aldrich) and IFN-γ (50 ng/mL; PEPROTECH) were added on days 4 or 6 for M1 activation, and differentiated macrophages were detached using accutase (STEMCELL Technologies) on days 6 or 8, respectively.

### Efferocytosis assay of human neutrophils and HUEhT in macrophages.

In total, 20,000 macrophages per well were seeded in a 96-well plate. After overnight culture, complete DMEM was replaced with serum-free DMEM before the introduction of neutrophils or HUEhT. Neutrophils isolated from healthy human donors and HUEhT were labeled with 10 μM CMFDA. Nonstimulated neutrophils, apoptotic neutrophils, TNF-α + healthy-IgG–treated neutrophils, ANCA-induced NETs, nonstimulated HUEhT, and necrotic HUEhT were washed, and NETs and necrotic HUEhT were exposed to PBS, CT-Ab (10 μg/mL), or anti-CD47 mAb (10 μg/mL) for 30 minutes. To address the role of FcR in efferocytosis of ANCA-induced NETs, macrophages were pretreated with FcR blocker (10 μg/mL; Invitrogen) or NETs were pretreated with anti-CD47 F(ab’)2 fragments for 30 minutes. After incubation, 100,000 neutrophils or 20,000 HUEhT detached using accutase were added to the macrophages and coincubated. Efferocytosis was assessed using still or time-lapse imaging. The percentage of CMFDA-labeled macrophages after 3 hours of coincubation and removal of the supernatant in the number of macrophages before introducing neutrophils was defined as the efferocytosis rate for still images. For time-lapse imaging, macrophages were labeled using PKH26 Red Fluorescent Cell Linker Kit (Sigma-Aldrich) before seeding, and the number of CMFDA-labeled cells in PKH26 labeled macrophages was evaluated. Images were captured every 7 minutes for 1 hour from the beginning of coculture. For assessing residual rate of NETs, 30,000 macrophages per well were seeded into 8-well chamber slides. PKH26-labeled NETs were exposed to PBS, CT-Ab, or anti-CD47 mAb for 30 minutes and then coincubated with macrophages for 3 hours. Slides were fixed with 4% PFA, covered with 1% BSA at room temperature for 1 hours, and exposed to anti-citH3 antibody (1:150; ab5103, Abcam). The sections were washed, incubated with Alexa Fluor 488 goat anti–rabbit IgG (Invitrogen), and mounted with VECTASHIELD with DAPI. The percentage of remaining NETs (PKH26^+^/citH3^+^/DAPI^+^ cells with 1 nucleus) in the number of engulfed NETs (PKH26^+^/citH3^+^/DAPI^+^ cells with multiple nuclei) was defined as residual rate of NETs.

### Quantitative PCR (qPCR) for human macrophages.

In total, 1,000,000 human nonstimulated neutrophils or NETs treated with CT-Ab or anti-CD47 mAb, and 200,000 macrophages were coincubated for 2 hours as described above. After removing the supernatant, total RNA was extracted using the RNeasy Mini Kit (Qiagen), according to the manufacturer’s instructions. A fixed amount of RNA was reverse transcribed into first-strand cDNA using SuperScript III (Invitrogen). qPCR was performed using Fast SYBR Green Master Mix (Thermo Fisher Scientific), according to the manufacturer’s instructions. The amount of transcripts was normalized to that of GAPDH.

The primers used were: *TNFA,* F: 5′-CTCTTCTGCCTGCTGCACTTTG-3′, R: 5′-ATGGGCTACAGGCTTGTCACTC-3′; *IL8*, F: 5′-GAGAGTGATTGAGAGTGGACCAC-3′, R: 5′-CACAACCCTCTGCACCCAGTTT-3′; *MCP1*, F: 5′-GCCTCCAGCATGAAAGTCTC-3′, R: 5′-AGATCTCCTTGGCCACAATG-3′; *IL1B*, F: 5′-CCACAGACCTTCCAGGAGAATG-3′, R: 5′-GTGCAGTTCAGTGATCGTACAGG-3′; and *GAPDH*, F: 5′-GGGAAGCTTGTCATCAATGGA-3′, R: 5′-TCTGGCTCCTGGAAGATGGT-3′.

### Animal models and treatment protocol.

SCG/Kj mice were purchased from the National Institute of Biomedical Innovation, Health, and Nutrition (Osaka, Japan). Eight-week-old mice were treated with i.p. injections of either 200 μg of anti-CD47 mAb (MIAP301, BioXcell) or rat IgG2a isotype CT-Ab (2A3, BioXcell) every 5 days for 2 weeks (*n* = 6 for each). Ten-week-old mice were then analyzed.

### Disease activity and severity in SCG/Kj mice.

For assessment of laboratory data, serum creatinine levels were determined using Creatinine kit (Serotec). Serum IgG levels were measured using IgG (total) Mouse Uncoated ELISA Kit (Thermo Fisher Scientific). The MPO-ANCA titer was determined by ELISA at the A-CLIP Institute (Chiba, Japan).

For histopathologic examination, fixed kidneys were embedded in paraffin and stained with H&E and periodic acid–Schiff (PAS). The sections for IHC staining were deparaffinized and rehydrated, followed by antigen retrieval using sodium citrate buffer (pH 6). Slides were probed with rat anti–mouse Ly6B antibody (1:100; MCA771G, Bio-Rad). For immunofluorescence staining, antigen retrieval was performed in sodium citrate buffer (pH 6) for CD68 staining. To confirm the presence of NETs, frozen kidney tissues were fixed with 4% PFA for 4 hours, dehydrated with 30% sucrose overnight at 4°C, frozen in OCT compound (Sakura), and cryosectioned into 5 μm–thick sections. The MPO and citH3–double-positive NETs were quantified using ImageJ software. Rat anti–mouse CD68 antibody (1:50; MCA1957GA, Bio-Rad), rabbit anti-citH3 antibody (1:100; ab5103, Abcam), or goat anti-MPO antibody (15 μg/mL; AF3667, R&D Systems) were used as the primary antibodies. Secondary antibodies included Alexa Fluor 488 goat anti–rat IgG (A11006, Invitrogen), Alexa Fluor 594 donkey anti–rabbit IgG (A21207, Invitrogen), and Alexa Fluor 488 donkey anti–goat IgG (A11055, Invitrogen). To assess the deposition of anti-CD47 mAb in glomeruli, specimens were immediately frozen in liquid nitrogen and then embedded in OCT compound. C57BL/6 mice treated with an i.p. injection of 200 μg anti-CD47 mAb were used as controls. Specimens were sliced into 5 μm sections using a cryostat and stained with Alexa Fluor 488 goat anti–rat IgG. To evaluate pathological changes in the kidney, glomerular activity (crescent formation, glomerular proliferation, and inflammatory cell infiltration) was calculated as previously reported (31). Sections were scored using a 0–3 scale for glomerular activity as follows: 0, no lesions; 1 = lesions in < 25% of glomeruli; 2 = lesions in 25%–50% of glomeruli; and 3, lesions in > 50% of glomeruli.

For FCM of splenocytes, the splenic cells were filtered through 100 μm cell strainers, washed, and filtered again through 70 μm cell strainers. RBC lysis was performed with RBC lysis buffer (Thermo Fisher Scientific). Aliquots of 5 × 10^5^ cells/tube were stained with the following antibody combinations: (a) Ly6C peridinin chlorophyll protein (PerCP)/Ly6G FITC/CD11b allophycocyanin (APC); (b) CD11c FITC/MHC-II APC/F4/80 PE; (c) CD3 PE; and d) B220 FITC.

For qPCR for mouse kidneys, renal tissue samples were stored in RNAlater solution (Invitrogen) until RNA extraction was performed. Total RNA was extracted, and first-strand cDNA was synthesized using 1 μg of total RNA, as described above. qPCR was performed using Fast SYBR Green Master Mix. The amount of transcript was normalized to that of 18S ribosomal RNA. The primers used were: *Mcp1*, F: 5′-TTAAAAACCTGGATCGGAACCAA-3′, R: 5′-GCATTAGCTTCAGATTTACGGGT-3′; *Ifng*, F: 5′-CAGCAACAGCAAGGCGAAAAAGG-3′, R: 5′-TTTCCGCTTCCTGAGGCTGGAT-3′; *Prf1*, F: 5′-GATGTGAACCCTAGGCCAGA-3′, R: 5′-GGTTTTTGTACCAGGCGAAA-3′; *Ifna*, F: 5′-CCTGAGAGAGAAGAAACACAGCC-3′, R: 5′-TTCTGCTCTGACCACCTCCC-3′; *Il1b*, F: 5′-TGGACCTTCCAGGATGAGGACA-3′, R: 5′-GTTCATCTCGGAGCCTGTAGTG-3′; *Nos2*, F: 5′-GAGACAGGGAAGTCTGAAGCAC-3′, R: 5′-CCAGCAGTAGTTGCTCCTCTTC-3′; *Cd206*, F: 5′-GTTCACCTGGAGTGATGGTTCTC-3′, R: 5′-AGGACATGCCAGGGTCACCTTT-3′; and *18s*, F: 5′-GCAATTATTCCCCATGAA-3′, R: 5′-AGGGCCTCACTAAACCAT-3′.

For RNA-Seq and data analysis, the quality of total kidney RNA was measured using a Bioanalyzer 2100 (Agilent). Libraries were generated using the NEBNext rRNA Depletion Kit (E6310; New England Biolabs) and NEBNext Ultra RNA Library Prep Kit for Illumina (E7530; New England Biolabs) according to the manufacturer’s instructions. The libraries were sequenced on a NovaSeq 6000 system (Illumina) by 2 × 150 bp paired-end settings. Raw sequencing reads with low quality and adapter sequences were removed or trimmed using Cutadapt v 2.10 (32). The trimmed reads were mapped to mm10 (Ensembl version 93) using STAR aligner v 2.5.1 (33). Differentially expressed genes (DEGs) were identified based on differences in expression levels (|log_2_ fold-change| > 1 and adjusted *P* < 0.05) between sample groups after removing genes with zero read count and non–protein-coding genes using DESeq2 v 1.28.1 (34). The Benjamini-Hochberg method was used to adjust the *P* value for multiple hypothesis testing. Canonical pathway analysis of DEGs was performed using IPA v 51963813 (QIAGEN Inc., https://www.qiagenbioinformatics.com/products/ingenuity-pathway-analysis/). BisqueRNA was used for bulk RNA-Seq deconvolution to estimate cell-type proportions (35). A single-cell RNA-Seq data set from the kidneys of C57BL/6 mice (18) was used as a reference. Although RNA-Seq was performed on whole-kidney RNA from 3 mice each, 1 RNA sample from anti-CD47 mAb–treated mice was excluded because of its poor RNA quality.

### Statistics.

The results obtained from in vitro and animal experiments are expressed as mean ± SEM and mean ± SD, respectively. Groups were compared using the 2-tailed Student’s *t* test for Gaussian distributions and Mann-Whitney *U* test for non-Gaussian distributions. Differences between greater than 2 groups were analyzed by 1-way ANOVA followed by Dunnett’s multiple-comparison test. *P* < 0.05 was considered significant. Statistical analysis was performed using JMP Pro version 14 (SAS Institute Inc.) and GraphPad Prism version 8.0.2 for Windows (GraphPad Software).

### Study approval.

Experiments using human materials were approved by the Sapporo City General Hospital Clinical Research Committee (approval no. R02-059-726). Animal experiments were approved by the Hokkaido University Animal Experiment Committee (approval no. 17-0016).

### Data availability.

The data generated are available from the corresponding author on reasonable request. RNA-Seq data were deposited in DDBJ (DRA014078). The Supporting Data Values are available in the supplement.

## Author contributions

SSA, DN, and AI contributed conceptualization, methodology, and formal analysis. SSA, MK, DN, and AI contributed data curation. SSA, DN, TK, MK, YU, KWK, SN, SI, TT, SM, UT, AI, and TA contributed writing of the original drafts. SI and TT contributed resources.

## Supplementary Material

Supplemental data

Supplemental video 1

Supplemental video 10

Supplemental video 2

Supplemental video 3

Supplemental video 4

Supplemental video 5

Supplemental video 6

Supplemental video 7

Supplemental video 8

Supplemental video 9

Supporting data values

## Version 1 06/27/2023

In-Press Preview

## Version 2 08/08/2023

Electronic publication
