# Supplementary material for: CD47 blockade ameliorates autoimmune vasculitis via efferocytosis of neutrophil extracellular traps
Source: JCI Insight. 2023 Aug 8;8(15):e167486. doi: 10.1172/jci.insight.167486 (PMC10445685; doi:10.1172/jci.insight.167486)
Supplement: Supplemental data [file jciinsight-8-167486-s056.pdf]

Supplementary Table S1. Patients' characteristics of AAV patients for renal histology

	MGA (N=8)	AAV (N=7)	LN class IV (N=4)	LN class V (N=4)
Age (year)	48.0 (36.3-65.0)	71.0 (67.0-74.0)	22.0 (15.0-33.5)	42.0 (28.0-49.3)
Male, n (%)	4 (50)	5 (71.4)	1 (25.0)	0 (0)
Serum creatinine (mg/dL)	0.67 (0.60-0.86)	1.13 (0.94-2.61)	0.80 (0.77-1.02)	0.54 (0.52-0.64)
MPO-ANCA (U/mL)	—	168 (41-290)	—	—
BVAS	—	12 (10-18)	—	—
Renal Pathology				
Global sclerosis (%)	12.0 (6.6-14.9)	14.3 (8.3-36.1)	0 (0-21.4)	8.89 (1.67-14.6)
Crescents (%)	0	25.0 (11.8-33.3)	3.70 (0.66-12.7)	0

Values for categorical variables are given as number (percentage); values for continuous variables are given as medians (interquartile ranges).
Abbreviations: MPO, myeloperoxidase; ANCA, anti-neutrophil cytoplasmic antibody; BVAS, Birmingham vasculitis activity score.

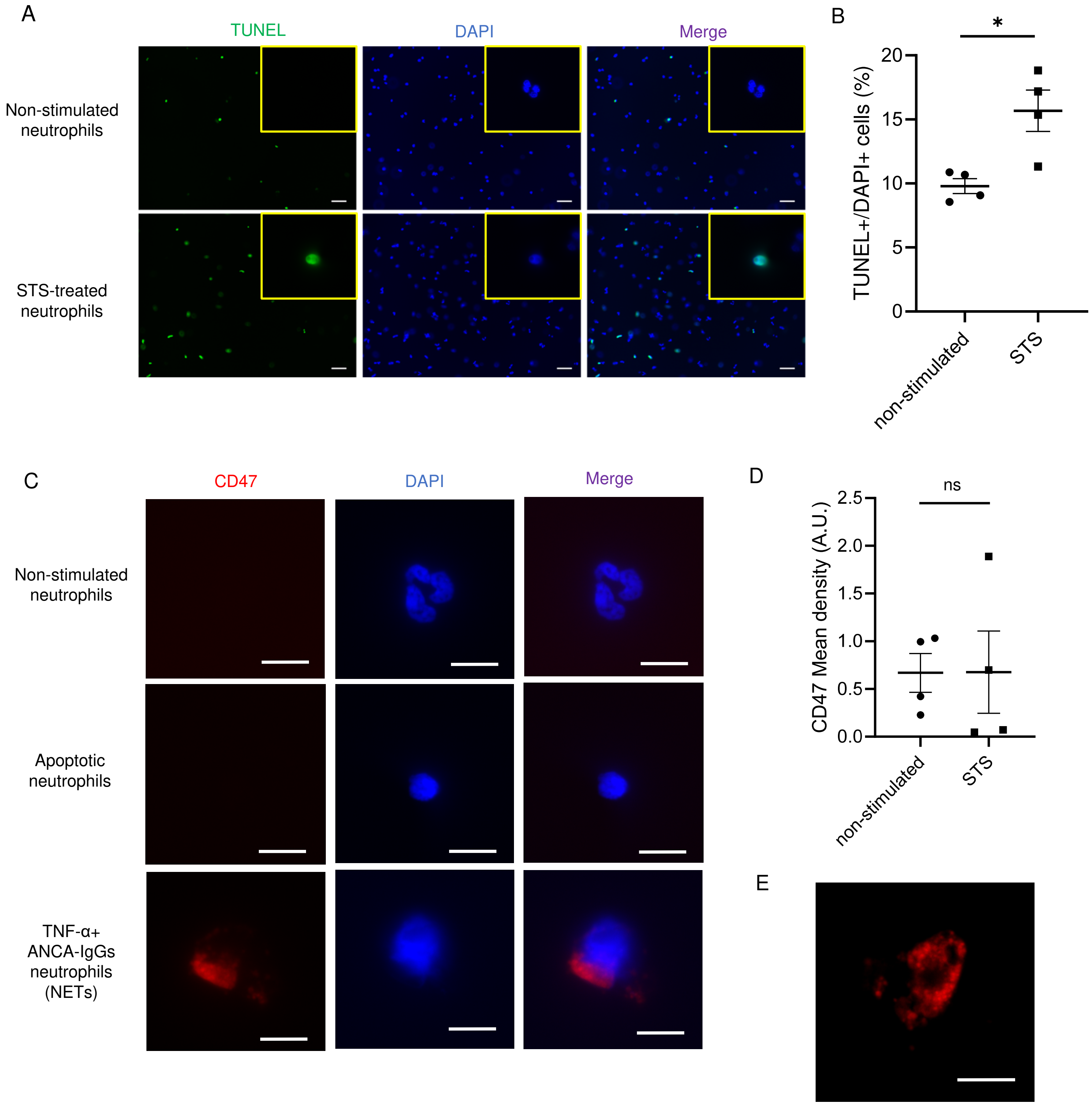

Supplementary figure S1. Apoptosis of neutrophils induced by STS. Apoptosis was induced using 0.05 µM STS. (A) Representative images of TUNEL staining and DAPI staining of neutrophils to evaluate apoptosis. Green: TUNEL staining; blue: DAPI staining. Scale bars: 50 µm. (B) Quantification of TUNEL-positive cells as a percentage of DAPI-positive cells (n=4 for each). (C) Representative images of CD47 and DAPI staining of neutrophils. Red: CD47; blue: DAPI staining. Scale bars: 10 µm. (D) Quantification of the mean density of CD47 on non-stimulated and apoptotic neutrophils (n=4 for each). (E) Representative images of CD47 staining of ANCA-induced NETs by optical sectioning microscopy. Red: CD47. Scale bars: 10 µm. Error bars represent SEM. *p<0.05, ns: not significant (Student's unpaired t-test).

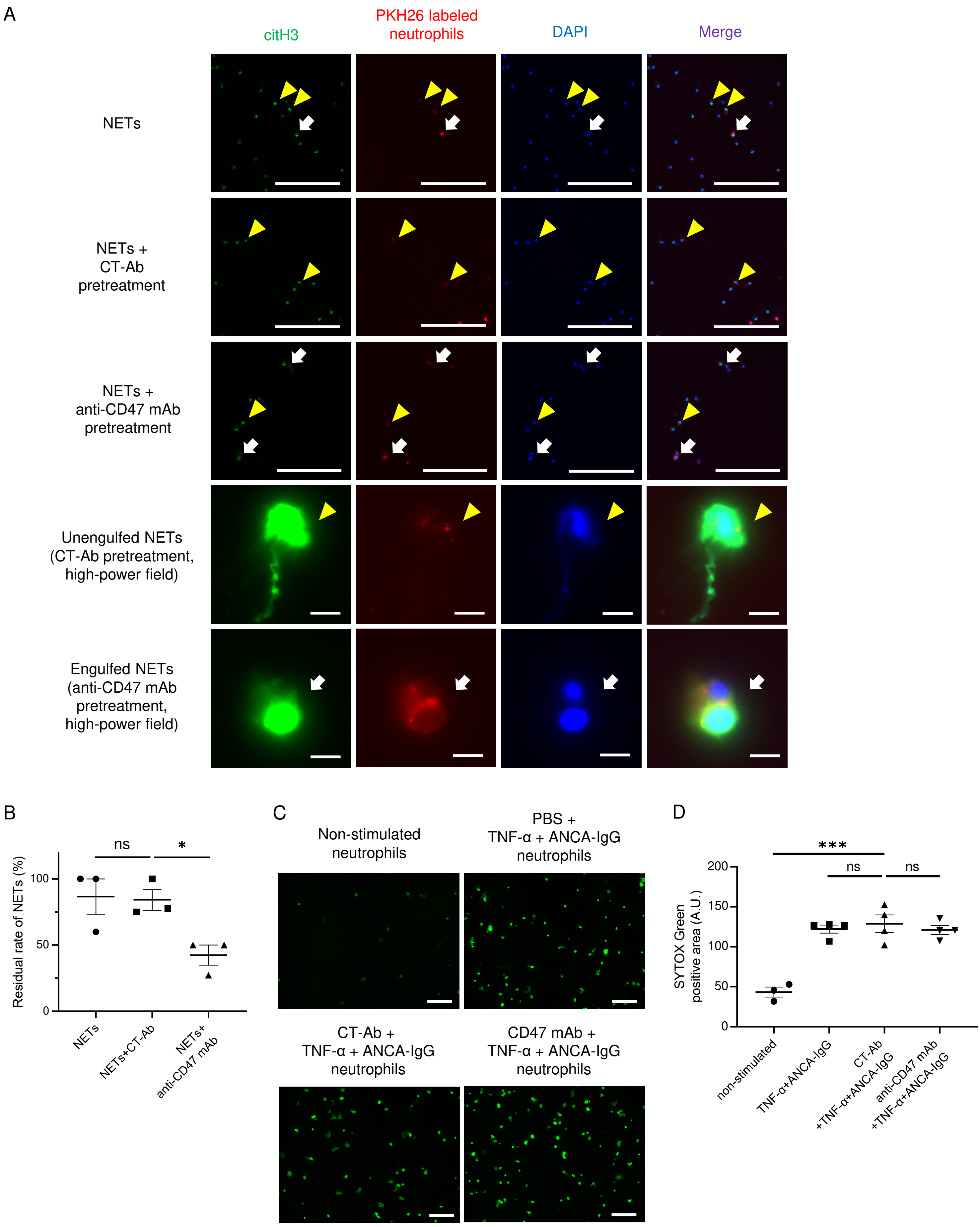

Supplementary Figure S2. The effect of CD47 blockade on NET formation. (A) Representative images of citH3, PKH26 labeled neutrophils and DAPI staining of efferocytosis assay to evaluate residual rate of NETs. Yellow arrowheads and white arrows indicated unengulfed and engulfed NETs, respectively. Green: citH3; red: PKH26; blue: DAPI staining. Scale bars: 200 µm (low magnification) and 10 µm (high magnification). (B) Residual rate of NETs (the percentage of unengulfed NETs) in A (n=3 for each). (C) NET formation in the presence of anti-CD47 mAb or CT-Ab was quantified by SYTOX Green staining. Scale bars: 200 µm. (D) Quantification of SYTOX Green-positive area of non-stimulated neutrophils (n=3), ANCA-induced NETs with PBS, and ANCA-induced NETs in the presence of CT-Ab or anti-CD47 mAb (n=4 for each). Error bars represent SEM. **p*<0.05, ****p*<0.001, ns: not significant (one-way ANOVA with post hoc Dunnett's multiple comparison test).

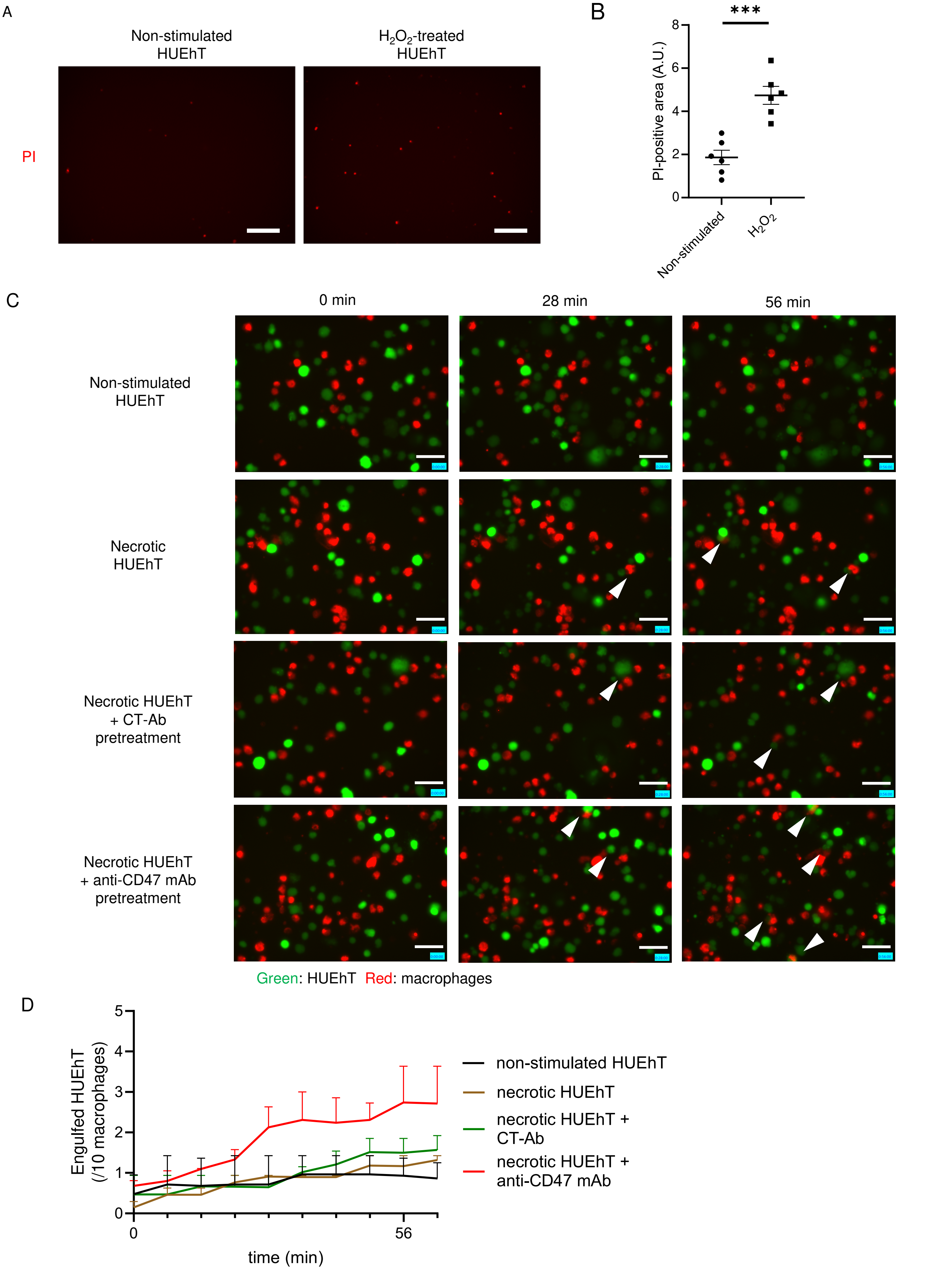

Supplementary figure S3. Efferocytosis of injured endothelium via CD47 signaling. Representative images (A) and quantitative analysis (B) of HUEhT by propidium iodide staining of non-stimulated and H_2O_2-treated HUEhT. Scale bars: 200 μm. (C) Representative time-lapse images of the efferocytosis assay of HUEhT. Scale bars: 50 μm. (D) Quantification of the number of engulfed non-stimulated HUEhT and H_2O_2-induced necrotic HUEhT cells treated with CT-Ab or anti-CD47 mAb (n=2 for each). Error bars represent SEM. ****p*<0.001 (Student's unpaired *t*-test).

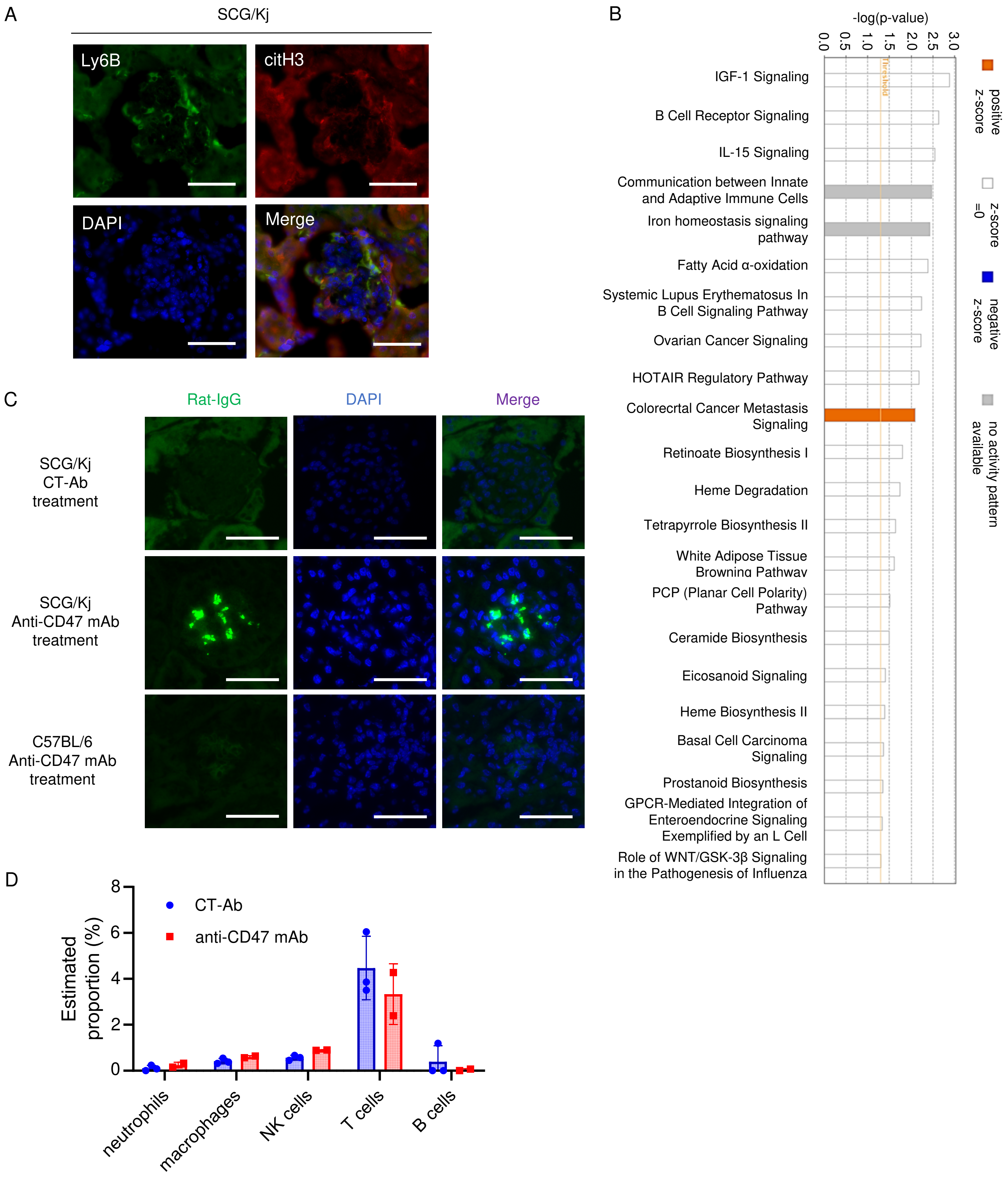

Supplementary figure S4. Effects of CD47 blockade on the kidneys of SCG/Kj mice. (A) Representative images of Ly6B, citH3, and DAPI staining of glomeruli of untreated SCG/Kj mice. Green: Ly6B; red: citH3; blue: DAPI staining. Scale bars: 50 μm. (B) Canonical pathways identified by IPA software in whole kidney. (C) Representative images of deposition of anti-rat IgG, which is the host species of anti-CD47 mAb. Scale bars: 50 μm. (D) Immune cell deconvolution results of whole kidney using Bisque.

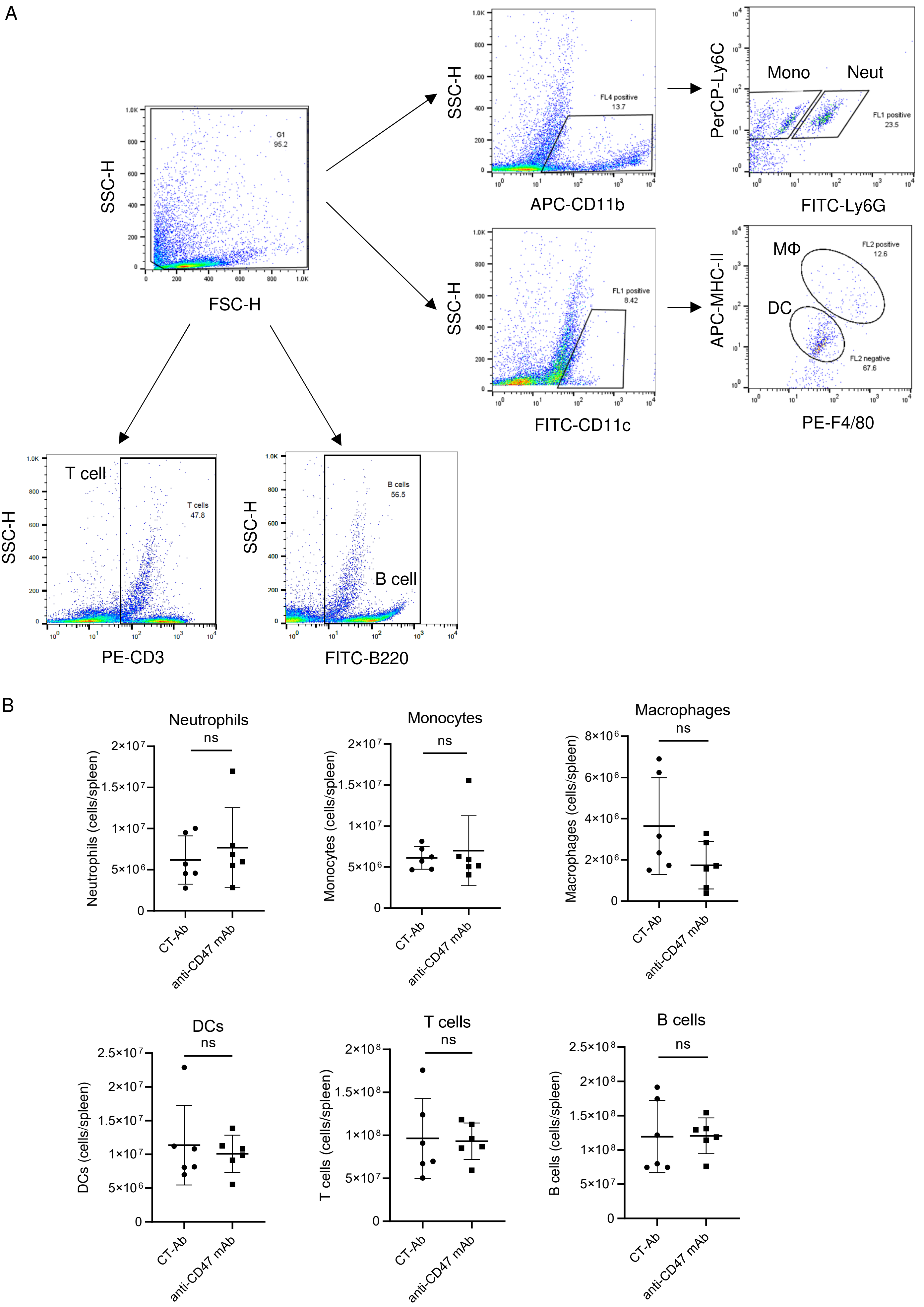

Supplementary figure S5. FCM of splenic immune cells from SCG/Kj mice. (A) Representative FCM plots for gating strategy to identify white blood cell differential counts in the spleens. (B) The number of neutrophils, monocytes, macrophages, DCs, T cells, and B cells of spleens. Error bars represent SD. ns: not significant (Student's unpaired *t*-test).

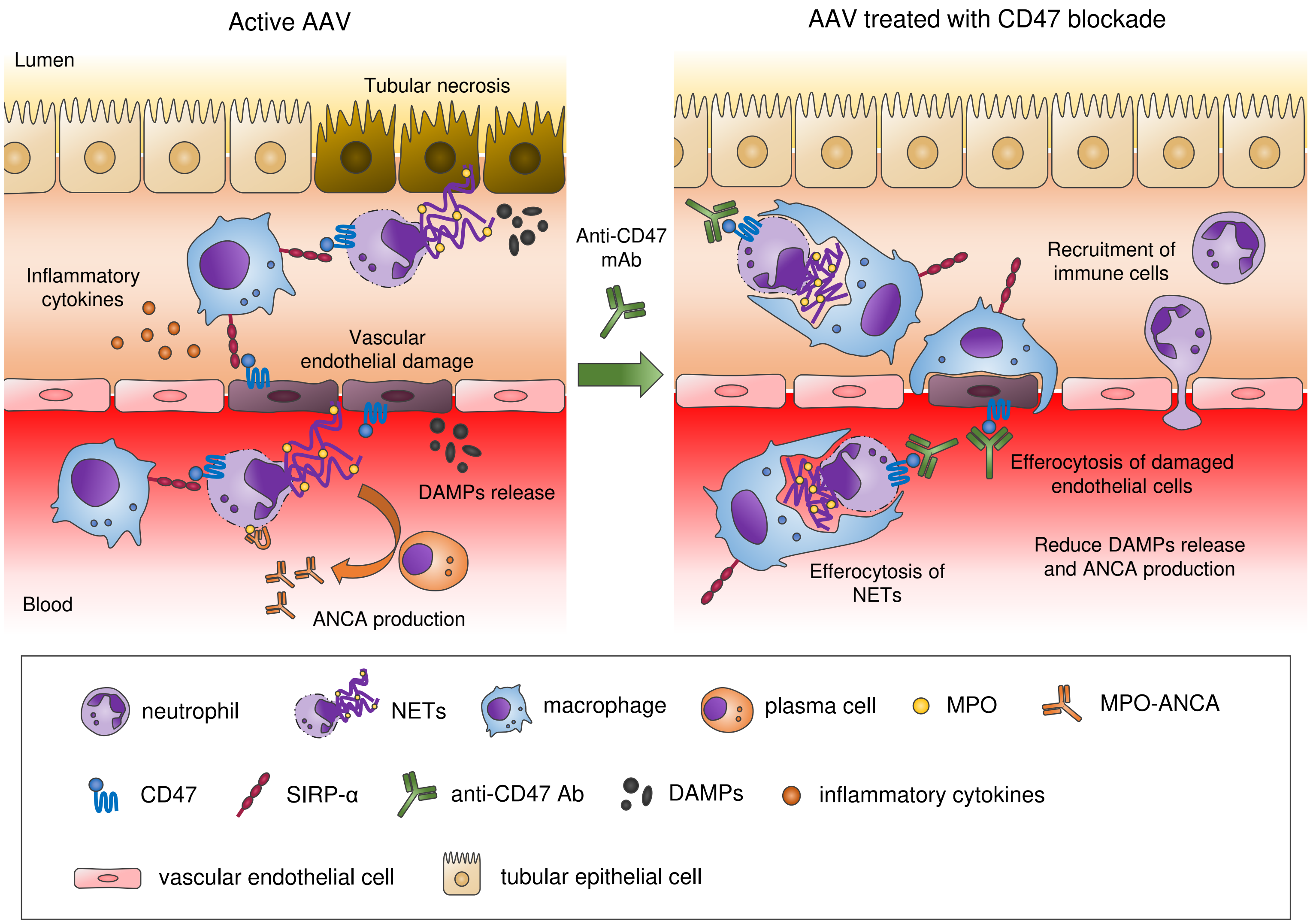

Supplementary figure S6. Working model for the role of CD47 in AAV. NETs and necrotic vascular endothelial cells overexpress CD47 to escape efferocytosis via CD47-SIRPα interactions. NET persistence contributes to endothelial injury and ANCA production. DAMPs released from necrotic cells induce inflammatory responses and organ damage. Treatment with anti-CD47 mAb promotes clearance of NETs and injured endothelial cells. Although activated macrophages induce neutrophil recruitment, CD47 blockade promotes efferocytosis of NETs and necrotic endothelial cells by macrophages, resulting in improved tissue injury and decreased ANCA production.
